# Fibrocytes in primary myelofibrosis

**DOI:** 10.18632/oncotarget.27971

**Published:** 2021-09-28

**Authors:** Kazuya Shimoda, Yoshinori Ozono, Kotaro Shide

**Keywords:** fibrocyte, monocyte, fibroblast, TGF-β, myeloproliferative neoplasms

Primary myelofibrosis (PMF) is a myeloproliferative neoplasm that is characterized by abnormal megakaryocyte and granulocyte proliferation and by progressive bone marrow (BM) fibrosis [1]. Its advanced phase is referred to as the overt fibrotic stage, and is characterized by constitutional symptoms such as fatigue, dyspnea, body weight loss, splenomegaly, anemia, leukoerythroblastosis in the peripheral blood (PB), and BM fibrosis.

More than 80% of PMF patients carry mutations that activate the JAK-STAT signaling pathway; about 50%, 25–30%, and 8% of PMF patients harbor *JAK2*V617F mutations, *CALR* exon9 frameshift mutations, and *myelo-proliferative leukemia virus oncogene* (*MPL*) mutations, respectively. These mutations lead to the autonomous activation of JAK-STAT signaling cascades without cytokine stimulation, resulting in autonomous cell growth. Ruxolitinib, a JAK2 inhibitor, was shown to drastically ameliorate constitutional symptoms and splenomegaly in patients with advanced PMF, but BM fibrosis was largely unchanged [2, 3].

The primary cell responsible for BM fibrosis in PMF remains obscure. Jacobson et al. studied a female patient with both A and B isoenzymes of glucose-6-phospatase dehydrogenase [4]. Only the type A enzyme was detected in PB granulocytes, erythrocytes, platelets, and BM cells, while equal proportions of A and B isoenzymes were observed in cultured BM fibroblasts; this indicated that BM fibroblasts in PMF did not originate from a neoplastic clone. As mesenchymal stromal cells (MSCs) produce collagen and fibronectin, BM fibrosis in PMF has been thought to be a reactive phenomenon caused by the overproduction of cytokines, including transforming growth factor (TGF)-β1, that are produced from megakaryocytes and platelets and that act on MSCs to produce collagen and fibronectin. The disappearance of thrombopoietin (TPO)-induced BM fibrosis following ablation of cells positive for Gli1 (a mesenchymal cell marker) also supports this theory [5]. On the other hand, Verstovsek et al. reported that fibrocytes were involved in BM fibrosis [6]. Fibrocytes are long, spindle-shaped, fibrocyte-like cells that differentiate from monocytes [7]. They produce collagen and fibronectin, and are positive for both hematopoietic cell markers and extracellular matrix proteins as alpha smooth muscle actin (αSMA) and vimentin, but negative for MSC markers such as CD90, Gli1, and leptin receptor (LepR). The BM of recipient mice transplanted with CD34^+^ cells from PMF patients exhibited fibrosis and a large number of collagen-producing cells. As these collagen-producing cells were positive for the human hematopoietic cell marker CD45, the cells primarily responsible for BM fibrosis in PMF were thought to be neoplastic fibrocytes rather than fibroblasts. In line with this, Maekawa et al. conducted a study using murine fibrocyte cell lines and TPO receptor agonist-induced murine MF model mice, and reported that TPO directly induced fibrocyte differentiation [8].

**Figure 1:**
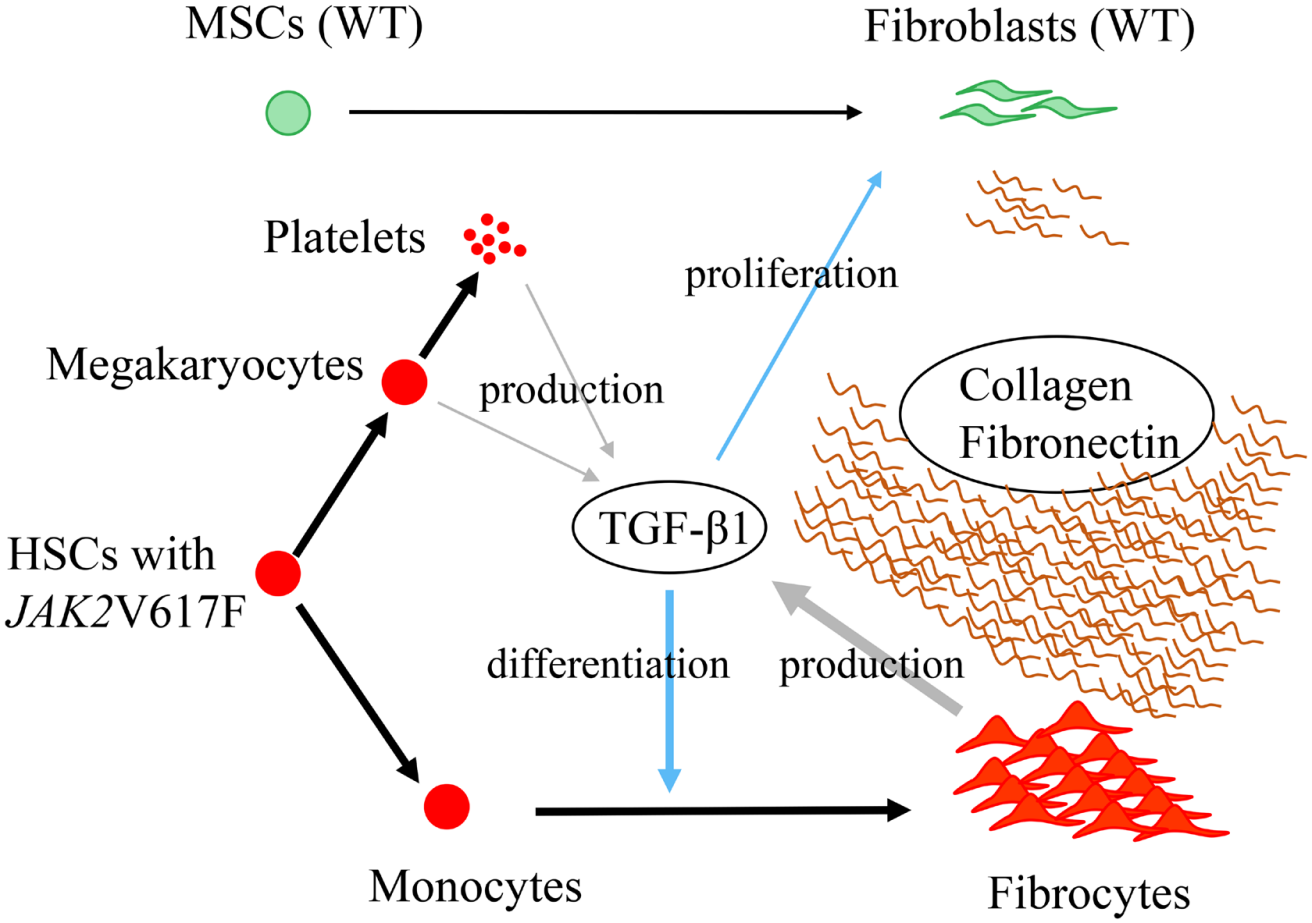
The mechanism of BM fibrosis in PMF (schematic diagram)(adopted from Figure 7 in Reference #9). Most collagen- and fibronectin-producing spindle-shaped cells in BM of *Jak2*V617F-induced PMF mice were neoplastic fibrocytes that had differentiated from neoplastic monocytes. Neoplastic fibrocytes produced TGF-β1, which supported their growth in an autocrine manner. MSCs: mesenchymal stromal cells, WT: wild type; HSCs: hematopoietic stem cells; TGF-β1: transforming growth factor β-1.

We recently reported that neoplastic fibrocytes were the major contributor to BM fibrosis in *Jak2*V617F-induced murine PMF model mice (Figure 1) [9]. One line of our previously reported *Jak2*V617F transgenic mice developed typical clinicopathological features of PMF, including anemia, leukoerythroblastosis in PB, massive splenomegaly, an increment of abnormal megakaryocytes, and progressive fibrosis in BM, and had reduced survival [10]. Five-day *in vitro* culture of BM cells under conditions that promoted differentiation of monocytes into fibrocytes demonstrated that dishes containing *Jak2*V617F BM cells exhibited many long, spindle-shaped cells compared with those containing wild-type (WT) BM cells. The spindle-shaped cells produced collagen and fibronectin, and while they were positive for both hematopoietic cell markers and extracellular matrix protein, they were negative for mesenchymal cell markers, indicating that the cells were fibrocytes. We next transplanted either *Jak2*V617F or WT BM cells (CD45.2), together with competitor WT BM cells (CD45.1), into irradiated recipient mice. Only recipient mice transplanted with the mixture of *Jak2*V617F and WT BM cells developed myelofibrosis, and the BM of these mice contained many collagen- and fibronectin-producing fibrocytes. Most of these fibrocytes were positive for CD45.2 but negative for CD45.1, indicating they originated from neoplastic clones. Compared to control mice whose BM cells consisted of less than 1% fibrocytes, neoplastic fibrocytes comprised 7–10% of BM cells in recipient mice transplanted with the mixture of *Jak2*V617F and WT BM cells. Fibroblasts expressing Gli1 and LepR comprised about 1% of the BM cells in these recipient mice.

We next ablated fibrocytes *in vivo*, and observed the effect on *Jak2*V617F-induced BM fibrosis. Fibrocyte depletion drastically reduced the numbers of reticulin and collagen fibers in BM from recipient mice transplanted with *Jak2*V617F BM cells, and also led to partial recovery of nucleated cells and erythroid progenitors in these mice, but had no effect on the increased number of hematopoietic stem cells and megakaryocytes. Accordingly, anemia improved, while thrombocytosis was minimally affected. The massive splenomegaly observed in recipient mice transplanted with *Jak2*V617F BM cells was ameliorated by fibrocyte depletion. The involvement of TGF-β1 in BM fibrosis was previously reported. Plasma TGF-β1 levels were about two-fold higher in recipient mice transplanted with *Jak2*V617F BM cells compared with control mice, and fibrocyte depletion normalized plasma TGF-β1 levels. We demonstrated that fibrocytes produced TGF-β1, and TGF-β1-neutralizing antibodies inhibited the differentiation of neoplastic monocytes into fibrocytes. These findings indicate that neoplastic fibrocytes differentiate from neoplastic monocytes in an autocrine manner in *Jak2*V617F-induced PMF. Our results are consistent with those of a previous study showing the usefulness of a therapy targeting fibrocytes [6]. In that study, xenograft mice transplanted with BM cells from PMF patients developed BM fibrosis, and treatment with the fibrocyte inhibitor serum amyloid P prolonged survival and slowed fibrosis development.

We demonstrated that neoplastic fibrocytes play an essential role in BM fibrosis in *Jak2*V617F-induced PMF; however, our findings could not rule out the involvement of fibroblasts in BM fibrosis. BM cells from *Jak2*V617F transgenic mice also consisted partially of collagen- and fibronectin-producing fibroblasts, in numbers that were 7–10 times greater than those in WT mice. In recipient mice transplanted with *Jak2*V617F BM cells, fibrocyte depletion also decreased the number of these fibroblasts, but only to a small degree. It is possible that these fibroblasts contribute slightly to BM fibrosis.

In summary, neoplastic fibrocytes, but not WT fibroblasts, were primary contributors to BM fibrosis in *Jak2*V617F-induced PMF.
